# The Adherence of Singaporean Students in Different Educational Institutions to National Food-Based Dietary Guidelines

**DOI:** 10.3390/nu12102995

**Published:** 2020-09-30

**Authors:** Mia Eng Tay, Emma Foster, Leo Stevenson, Iain Brownlee

**Affiliations:** 1 Newcastle Research and Innovation Institute, Devan Nair Building, Singapore 600201, Singapore; tay_mia_eng@nyp.edu.sg (M.E.T.); stevenl2@hope.ac.uk (L.S.); 2 School of Chemical & Life Sciences, Nanyang Polytechnic, Singapore 569830, Singapore; 3 Human Nutrition Research Centre, Population Health Sciences Institute, Newcastle University, Newcastle upon Tyne NE2 4HH, UK; dremmafoster@outlook.com; 4 School of Health Sciences, Liverpool Hope University, Hope Park, Liverpool L16 9JD, UK; 5 Faculty of Health and Life Sciences, Northumbria University, Newcastle-upon-Tyne NE1 8ST, UK

**Keywords:** food-based dietary guidelines, diet quality, salt intake, fruit and vegetable consumption

## Abstract

There are currently limited data on the dietary habits of young Singaporeans. This study aimed to evaluate the adherence of 17–21 year olds attending different educational institutions using a novel diet-quality scoring method. Dietary data were collected using a single weekday 24 h dietary recall in a cross section of 536 Singaporeans aged 17–21 years. An 11 category scoring system (0.0–100.0) was used to define adherence to food based dietary guidelines. Demographic and self-reported data were also collected via a questionnaire, BMI status, and using Mann-Whitney and Kruskal-Wallis (non-parametric) tests, with post-hoc Bonferroni-corrected tests. The median diet quality score was 48.5 (IQR 40.5, 56.4) for this cohort, with component scores for “Total fruit”, “Whole fruit”, “Total vegetables”, “Dark green leafy & orange vegetables”, “Whole grains”, “Dairy products”, and “Sodium” frequently scoring the minimum value. Median diet quality scores were statistically different for groups by ethnic origin (*p* < 0.001) and by educational institution (*p* < 0.001). Intake of fruit, vegetables, and whole grains is minimal, while sodium intake is frequently too high in young Singaporeans. Differences across ethnic groups and types of educational institutions suggest the need for targeted interventions to improve dietary habits in this population.

## 1. Introduction

Adolescence is the transitional stage that lies between childhood and adulthood and has previously been defined as the time between 10–19 years old [1]. The transition from adolescence to adulthood is a period of the life course where there is a rapid change in nutritional requirements [2]; social, physical, and environmental influences [3,4]; and often increased independence in decision-making, including decisions that relate to dietary habits and lifestyle [5,6]. This period of transition has been suggested to be important in developing dietary habits that may track into later life [7], thereby affecting lifelong disease trajectory [8,9,10]. Previous reports suggest that dietary habits in adolescent populations are frequently sub-optimal with a high intake of saturated and total fat, but a low intake of fruit, vegetables, fibre, and calcium-rich foods [11,12,13].

Singapore is an island nation that has rapidly developed a world-class reputation in education [14]. The majority of Singaporeans are from three major ethnic groups (Chinese, Malay, and Indian) which form the core of multicultural culinary offerings in Singapore [15]. Existing data from adult cross-sectional studies suggest that ethnicity is linked to divergent dietary habits [16] and health outcomes [17,18]. Nationally-representative dietary data have been collected in Singaporean adults (aged 18 to 69 years) within the National Nutrition Study (NNS) since 1993 [19] using food frequency questionnaire methods. However, dietary habits specific to students have only been collected previously as part of the Students’ Health Survey, using a short-form dietary questionnaire [20]. As such, information on current dietary habits in Singaporean adolescents is limited, particularly in relation to the educational institution that they are attending.

Within the Singaporean education system, almost all adolescents (at the age of approximately 17 years) enter post-secondary education into one of three types of institutes: an Institute of Technical Education (ITE—23.7% of all individuals after secondary school completion), a polytechnic (47.8%), or a junior college (28%) [21]. Each type of educational institution has specific core pedagogic missions. It is also important to note that only junior colleges are monitored by the Ministry of Health for the standards of food made available on-campus [22,23].

Approaches to estimate the adherence of individuals to notional ideals of dietary habits have been utilized more frequently in nutrition research since the 1990s, with earlier iterations in existence since the 1940s [24,25]. Such methodologies frequently compare estimates of dietary intake against food or nutrient based dietary guidelines [26,27], providing a single numeric indicator from complex dietary data [28,29]. Such approaches have been used to provide feedback to individuals on overall dietary habits [30,31] and could help focus future public health efforts for specific populations [32]. Such approaches may also be less prone to confounding factors than an evaluation of intake of single or multiple nutrients and food groups [33]. While approaches to estimate overall dietary quality have been published for Singaporean infants and children [34,35], the authors believe that no such method has currently been defined for late adolescents and young adults.

This study therefore aimed to fill current gaps in knowledge by assessing dietary habits (on school days) in a cross-section of Singaporean late adolescents/early adults attending the three main types of post-secondary educational institution. In order to do this, a novel diet quality scoring approach was developed to assess the adherence of individuals in this cohort to Singaporean food-based dietary guidelines, as estimated through a single weekday 24 h dietary recall.

## 2. Materials and Methods

The study method was approved by the Ethics Committee (Faculty of Science, Agriculture, and Engineering), Newcastle University on the 24 October 2014 and Institutional Review Board and Nanyang Polytechnic (NYP IRB Ref: SCL-2014-001) on the 18 September 2014. As the participants included students from a junior college, additional approval from the Ministry of Education, Singapore was obtained on the 23 February 2015. Email approval was obtained from the principal of ITE College Central on 19 March 2015.

The eligible target population was Singaporean nationals, aged 17–21 years (the standard age range in which individuals attend post-secondary education). Participants of Chinese, Malay, and Indian ethnic origin were subsequently recruited via school portals and posters. Posters were displayed on students’ notice boards for the attention of students and to encourage word of mouth recruitment through friends. On-site recruitment was also performed where responses from school portal or posters was low. Interested participants contacted the research lead (M.E.T.) and received additional information on the project prior to collection of informed consent. For participants aged 17 years, parental consent was also obtained. Following this, a separate participant data form was developed to collect details of their name, contact details, address, ethnicity, sex, date of birth, education institute, self-reported weight, and height. Two separate recruitment drives were undertaken. The first recruited students were from the polytechnic site only, using a purposeful sampling approach to ensure inclusion of adequate numbers of individuals by ethnic origin and sex. This approach ensured an adequate representation of participants of Malay and Indian ethnic origin and increased the number of male participants. The second recruited an additional 100 individuals from the Institute of Technical Education and the Junior College by convenience sampling (i.e., all individuals who agreed to take part were recruited) to allow comparisons between institutions. Data were from a total of 536 participants (collected/recruited between November 2014 and August 2015). The most conservative estimate of a representative sample from a population of approximately 100,000 individuals [21] with 95% chance of estimating the true population mean and desired accuracy within 5% would require a total of 383 participants [36]. Additional individuals were recruited to help ensure additional statistical power for sub-analyses within the time constraints of the proposed study.

The 24 h recall form was adapted for use from The UK Low Income Diet and Nutrition Survey [37]. A multiple-pass approach was taken to collecting dietary data from participants. This approach was adapted from the USDA 5-step multiple-pass method [38,39] to help improve the accuracy of the dietary recall [40]. Data were collected by a trained researcher at the student’s particular educational institute. Model plates, bowls, and cutlery alongside a compendium of local food pictures [41] were developed to improve the quality of the portion size estimation by the participants. Food composition data were collated from local tables as well as international tables (Malaysia, Australia, and UK) as previously described [34].

The scoring system for the Healthy Eating Index for Singaporean adolescents (HEI-SGA) was based on similar approaches used to design the Healthy Eating Index 2010 [42,43] and the Healthy Eating Index for pregnant women in Singapore, HEI-SGP [44], but modified according to Singaporean food-based dietary guidelines for individuals of this age range [45]. The Singaporean Health Promotion Board launched My Healthy Plate in 2014 in order to better communicate the stipulated dietary guidelines [45]. The current approach to assess adherence to these guidelines included 11 components (presented in Table 1 below).

A score for each component was calculated based on Singapore’s My Healthy Plate and dietary guidelines and adjusted based on recommended energy intake for individuals of that particular sex and age [45,46]. For example, if an individual was recommended to consume 2 servings of fruit with a total dietary energy intake of 2300 kcal diet/day, the maximum standard for the “Total fruit” (i.e., all forms including juice) component was calculated as ≥0.87 servings/1000 kcal diet. Zero points were allocated if no fruit in any form was consumed, while a maximum of 5 points were allocated if more than 0.87 servings of fruit per 1000 kcal were consumed. The sum of all component scores was then divided by 90 and multiplied by 100 to give a total score that could hypothetically range from 0–100.

All statistical analyses were performed using the Statistical Package for Social Sciences, SPSS, version 26.0 for Windows (IBM Corp., Armonk, NY, USA) and statistical significance for all the tests was defined at *p*-value < 0.05. Total HEI-SGA scores for the cohort were parametrically distributed, but sub-groups were not. As all component scores were non-parametric, it was decided to carry out comparisons between groups using Mann-Whitney and Kruskal-Wallis (non-parametric) tests, with post-hoc Bonferroni-corrected tests.

## 3. Results

Complete 24 h food recall and questionnaire data were collected for all participants. Overall, the median HEI-SGA score was low at 48.5 (IQR 40.5, 56.4) out of 100. Component scores for “Total fruit”, “Whole fruit”, “Total vegetables”, “Dark green leafy & orange vegetables”, “Whole grains”, “Dairy products”, and “Sodium” were frequently zero or close to zero within this cohort, while component scores for “Total rice and alternatives”, “Total protein foods”, “Total fat”, and “Saturated fat” were towards maximal for the majority of the population (see Table 2 for additional detail). Male (median 48.2, IQR 40.1–56.4) and female (48.8, 42.1–56.4) participants had similar total HEI-SGA scores (*p* = 0.883), with female participants scoring statistically higher component scores for “Whole fruit”, “Total vegetables”, “Dark green leafy & orange vegetables”, and “Total rice and alternatives” when compared by independent sample Mann Whitney U test (*p* < 0.05, see Table 2) despite similar median values. A higher proportion of males appeared to score a maximum score for the “Total protein foods” category (*p* < 0.001), although again, median scores were similar (males median = 10, IQR 9.2–10.0 vs. females 10.0, 5.7–10.0), see Table 2).

There was no significant difference among the median total HEI-SGA and component scores for different categories of BMI (see Table 3), but the highest BMI category group appeared to consume fewer energy-adjusted portions of “Rice and alternatives” and “Dairy and alternatives” compared to other groups (*p* = 0.007 and 0.008, respectively).

Table 4 and Table 5 highlight that the majority of component scores differed across ethnic groups and educational institutions. Participants of Chinese ethnic origin had the statistically highest (*p* < 0.001) Total HEI-SGA score (median 52.4, IQR 44.4–60.4) followed by those of Indian (47.6 38.5–54.9) and Malay (44.4, 37.2–50.2) ethnic origin (see Table 4). Students from the Junior College had a statistically higher (*p* < 0.001) Total HEI-SGA score (56.6, 48.1–64.4) than those attending the polytechnic (47.4, 38.2–54.7) or ITE (47.4, 40.2–52.6). Junior College students appeared to have markedly higher median scores for “Total fruit”, “Whole Fruit”, and “Total vegetables” than students from other educational institutions (see Table 5 for further detail).

## 4. Discussion

With accelerated economic development and urbanization over the past decades, Singapore faces current and future public health challenges with non-communicable diseases [47], despite having one of the highest estimates of healthy life expectancy of any country or territory globally [48]. The use of diet quality indices has allowed researchers to consider overall dietary habits in relation to measures of a population’s health using a single useful indicator with varying degrees of complexity [42]. The authors believe that the approach described in this paper provides a rational means to look at overall dietary habits in this population group. As information of dietary intake within Singaporean late adolescents/early adults is extremely limited, the current dataset should also provide support to future national public health efforts. The approaches taken to consider how educational institution and other factors are associated with diet quality in a diverse cross-section may have wider applications for similar future studies globally.

The HEI-SGA scores across the cohort suggested that dietary intake was frequently divergent from dietary guidelines in this cohort, with the median score of the current sample (48.5 out of 100) appearing lower than similar estimates of diet quality in Singaporean pre-teen (median 65.4 out of 100) and infants (mean 44.2 out of 65) noted in recent studies [34,35].

While wider data on dietary habits in late adolescents/young adults remain limited, previous studies have suggested similar findings within individuals of this age range elsewhere in the world. Cross-sectional data from the UK National Diet and Nutrition Survey highlight that diet quality is far from ideal within this age range [49,50], with US cross-sectional data also highlighting that individuals aged 14–18 years tended to have lower diet quality estimates than younger children [51,52]. Analysis of the Norwegian Longitudinal Health Behaviour Study dataset (which includes dietary data collection from a Norwegian longitudinal cohort at eight time-points between 14 and 30 years) highlighted a dip in fruit and vegetable consumption in early adulthood (until age 21 years and 23 years, respectively), alongside an increased intake of sugar-sweetened beverages and confectionary items between the ages of 14 years and 18 years [53]. A similar study in the US suggested that the diet quality of individuals may improve modestly between the ages of 16 years and 20 years [54].

The component scores that most frequently scored highly (i.e., individuals met or exceeded dietary guidelines) were for “Total rice and alternatives” and “Total protein foods”. These findings were similar to previous studies, where intake of carbohydrates and proteins in late adolescents and early adults in developed countries was rarely below the recommendation [55,56]. Although almost all participants met or exceeded “Total rice & alternatives” recommendations, the component score for “Whole grains” was negligible across the cohort. This somewhat aligns with data on adult intake (aged 18 to 69 years) from the Singapore National Nutrition Survey (NNS) conducted in 2010, where it was noted that only 27% of Singaporeans consumed one serving or more of wholegrain products per day [19], up from 8.4% in 2004. O’Neil et al. (2011) reviewed the consumption of whole grains in USA children and adolescents using the National Health and Nutrition Examination Survey (NHANES) 1999–2004 [57]. It was concluded that the consumption of whole grains was low, with a mean serving of 0.63 servings of whole grains/d for adolescents, aged 13–18 years. Factors that have been suggested to drive low intake of whole grains within this age group include poor expected palatability, limited availability outside of the home, and consumers’ inability to identify wholegrain products [58,59]. There has been increased public health promotion of wholegrain consumption in Singapore, including increasing the availability of whole grains by working with the food manufacturers to produce more whole grain products and actively broadcasting the benefits of whole grains through initiatives such as supermarket tours and school talks (Health Hub, 2017). In 2016 (after the end of data collection for the current study), a major shift was made in the Heathy Meals in Schools Programme to stipulate that at least 20% of the rice or alternative cereal-based foods should be whole grains and only wholemeal bread can be used to prepare the sandwiches [22]. However, this programme is not mandatory for all post-secondary education establishments. Currently, only food provision at Junior Colleges falls under the purview of the Ministry of Education guidelines. Evaluation of whether this update in recommended food provision has increased wholegrain food intake in Junior College students would be interesting and should be possible through collection of further dietary data in this population.

The median component scores for the “Total fruit”, “Whole fruit”, “Total vegetables”, and “Dark green leafy & orange vegetables” components were also low across the cohort. Data from the Singaporean National Nutrition Survey suggests that intake of fruit and vegetables may have gone down in adults over time, with a lower percentage of individuals meeting fruit and vegetable recommendations in 2010 versus 2004. The intake of fruit is lowest in 18–29 year-olds, but vegetable intake tends to be higher both for males and females in this age range than for older groups [19]. Low intake of fruit and vegetables appears relatively common in late adolescents/early adults in many parts of the world [13]. For example, a recent study conducted in India found that adolescent girls’ consumption of vegetables and fruit was also considerably below the national Recommended Dietary Intake [60].

The approach taken here was based on the wording of the food-based dietary guidelines in Singapore. Weighting was used within scoring categories to ensure that intake of specific items (e.g., whole fruits and green leafy and orange vegetables) was included in the criteria for maximal scoring. Individuals who scored high for “Whole fruit” and “Dark green leafy & orange vegetables” would also score highly for “Total fruit” and “Total vegetables”. While the current approach aligns well with food-based dietary guidelines, an alternative scoring approach could have been to limit the number of servings (of, for example, fruit juices or smoothies) that could be credited with a score. Due to the low intake of fruits and vegetables in the current cross-section (>60% of all participants scored zero for all fruit and vegetable component scores), this appears unlikely to have affected the overall findings of the current study.

The lowest-scoring nutrient-based category in the HEI-SGA was sodium, for which the majority of individuals scored less than 1.5 out of 10. The high sodium intake could possibly be attributed to the frequent consumption of out-of-home food consumption previously noted in Singapore [61], where many popular dishes (both of Asian and Western origin) tend to have high sodium content [62]. While attempts were made to estimate total salt (including elective salt) consumption accurately during collection of 24 h recall information, previous studies would suggest that total salt intake may be under-reported using such methods [63].

It appears that the dietary habits among the students attending Junior College were closer to the ideal. Students attending this institution tend to start and end the school day earlier compared to the Polytechnic and ITE students. This could be driven by confounding factors like socio-economic status linked to educational attainment [64,65] that have not been collected within the current study. It is also unclear whether on-campus food provision was a major driver for more or less positive dietary habits. Our current analysis has not separated site of food consumption beyond whether items were consumed within the home and out-of-home, but this would form a rational focus for future research.

The HEI-SGA provides an approach to systematically evaluate the diet quality of Singaporean late adolescents/early adults against the Health Promotion Board’s recommendations. The method used to estimate HEI-SGA scores was largely based on the previous HEI-2010 method but was adapted to Singaporean dietary guidelines. This previous method included energy adjustments for each component score. Due to the potential for the methods for dietary intake estimation (24 h food recall) to under-report intake, the authors felt that energy adjustment would help mitigate these potential limitations [41,43]. It would have been more ideal to estimate physical activity levels in this cohort to better define target energy intake [34]. However, the design of the current study did not allow this. Estimation of physical activity energy expenditure is particularly relevant for similar future studies where guidelines for total dietary energy intake differ based on physical activity levels.

Weight and height of the respondents were obtained based on self-declaration. This approach is not as accurate as direct measurement methods [66] and may skew the HEI-SGA scoring for under- and over-reporters. The proportion of individuals in this cohort who were self-reported as high risk/obese (10.4%) was similar to the proportion recently estimated to exist in the adult population (8.7%) in Singapore [17]. A novel food atlas was developed for culturally-relevant food items in Singapore and used to support the collection of dietary recall data [41]. While similar tools have been used in other populations effectively [67,68], it must be noted that the current tool has not been validated. Nonetheless, the authors believe that this approach helped to ensure better estimation of food portion sizes by respondents, thereby benefitting the overarching study outcomes. Ideally, dietary data collection would also have involved replicate collection across 3–4 days [69]. However, neither direct measurement of height and weight or additional dietary data collection were possible within the time scale and were not available resources of the current study. Due to the scarcity of data of dietary habits in Singaporeans of this age, it was decided to recruit a larger and more representative cohort for this cross-sectional study. The design of this study aimed to evaluate the dietary habits of this population in relation to the educational institution setting and so only dietary recall data from weekdays was collected. While the current study had a sample size that would be likely to adequately represent the overall population of post-secondary Singaporean students, the sub-analyses carried out here on sex, ethnicity, BMI status, and institution of study may not have been adequately powered. The current comparison only included data from three specific institutions that may not have represented the wider range of educational institutions in Singapore. Future studies should consider more extensive sampling across a wider range of institutions and advertising for participants through more inclusive and widely-accessed methods (like institutional emails or social media platforms). Repeated and/or more objective approaches for dietary data collection like weighed food diaries and height and weight measurements should be measured directly by future study teams to improve confidence in dietary and body weight status data. Additional record-keeping of individuals who declined participation or withdrew would help align with consensus guidelines on best practice (see Appendix A) for the running of observational studies [70].

Many savoury food items consumed by participants (e.g., fried rice, stir-fried noodles, and curry chicken) contained proportions of food items from multiple food groups. Estimation of the contribution of these items to the intake of each food group required utilization of available recipes and thus, may not accurately depict the actual food consumed.

There appears to be a future research need to develop interventions (for instance, to encourage fruit and vegetables consumption) for this targeted group of post secondary school students over a period of time and then to review the impact of the intervention by calculating and comparing the HEI-SG before and after the interventions. The multi-vendor nature of each cafeteria/eatery in Singaporean educational institutions reduces potential issues of access to positive choices [22]. Our findings suggest that such interventions may need to focus on improving personal choice of food items towards better meeting food-based dietary guidelines. The existing standards for more prudent food provision (currently recommended/enforced at Junior Colleges) could be considered at both polytechnics and ITEs.

## 5. Conclusions

This work proposes a means of assessing diet quality in Singaporean late adolescents/young adults and also highlights some of the major areas for improvement in the diet for this population. Public health strategies should be customized to address the low intake of fruit and vegetables, whole grains, and dairy products and the high intake of sodium for this group of adolescents, with particular consideration for approaches that effectively engage students at different types of educational institutions and from different ethnic groups.

## Figures and Tables

**Table 1:** Scoring elements used to calculate the Healthy Eating Index for Singaporean Adolescents (HEI-SGA).

No.	Component	Standards for Minimum Score of Zero	Standards for Maximum Score	Maximum Score
1	Total fruit	No fruit	≥0.87 serves/1000 kcal	5
2	Whole fruit	No whole fruit	≥0.43 serves/1000 kcal	5
3	Total vegetables	No vegetables	≥0.87 serves/1000 kcal	5
4	Dark green leafy & orange vegetables	No dark green leafy and orange vegetables	≥0.43 serves/1000 kcal	5
5	Whole grains	No whole grains	≥1.30 serves/1000 kcal	10
6	Dairy and alternatives	No dairy and alternatives	≥0.43 serves/1000 kcal	10
7	Total protein foods	No protein food	≥1.08 serves/1000 kcal	10
8	Total rice & alternatives	No rice and alternatives	≥3.04 serves/1000 kcal	10
9	Total fat	≥40% of energy	≤30% of energy	10
10	Saturated fat	≥20% of energy	≤10% of energy	10
11	Sodium	≥870 mg/1000 kcal	≤435 mg/1000 kcal	10
-	TOTAL	-	-	90

**Table 2:** Median component and total HEI-SGA values across all participants and by sex.

	All (*n* = 536)	Female (*n* = 304)	Male (*n* = 232)	
Components	Median (IQR)	Median (IQR)	Median (IQR)	* *p*-Value
Total fruit	0.0 (0.0, 4.1)	0.0 (0.0, 4.8)	0.0 (0.0, 3.2)	0.001
Whole fruit	0.0 (0.0, 5.0)	0.0 (0.0, 5.0)	0.0 (0.0, 0.0)	<0.001
Total vegetables	0.6 (0.0, 2.4)	0.9 (0.0, 2.7)	0.3 (0.0, 1.7)	0.014
DGLOV	0.0 (0.0, 0.0)	0.0 (0.0, 0.0)	0.0 (0.0, 0.0)	0.009
Total rice & alternatives	10.0 (7.9, 10.0)	10.0 (7.4, 10.0)	10.0 (8.5, 10.0)	0.013
Whole grains	0.0 (0.0, 0.0)	0.0 (0.0, 0.0)	0.0 (0.0, 0.0)	0.956
Dairy and alternatives	1.9 (0.0, 7.0)	1.9 (0.0, 7.5)	2.1 (0.0, 6.4)	0.811
Total protein foods	10 (6.9, 10.0)	10.0 (5.7, 10.0)	10.0 (9.2, 10.0)	<0.001
Total Fat	10 (8.4, 10.0)	10.0 (8.0, 10.0)	10.0 (8.8, 10.0)	0.621
Saturated fat	10.0 (7.1, 10.0)	9.8 (6.9, 10.0)	10.0 (7.4, 10.0)	0.097
Sodium	0.0 (0.0, 0.0)	0.0 (0.0, 0.0)	0.0 (0.0, 0.0)	0.722
Total HEI-SGA score	48.5 (40.5, 56.4)	48.2 (40.1, 56.4)	48.8 (42.1, 56.4)	0.883

* Based on independent sample Mann-Whitney U Tests between males and females. DGLOV—dark green leafy and orange vegetables, HEI-SGA—Healthy Eating Index for Singaporean adolescents, IQR—diet quality score.

**Table 3:** Median component and total HEI-SGA values across all participants and by BMI category.

	Median (IQR) Component or Total Score				
	At Risk of Nutrient Deficiency (*n* = 106)	Healthy (*n* = 281)	Moderate Risk (*n* = 95)	High Risk (*n* = 54)	* *p*-Value
Total fruit	0.0 (0.0, 3.5)	0.0 (0.0, 4.2)	0.0 (0.0, 4.8)	0.0 (0.0, 4.1)	0.757
Whole fruit	0.0 (0.0, 3.9)	0.0 (0.0, 5.0)	0.0 (0.0, 3.8)	0.0 (0.0, 5.0)	0.349
Total vegetables	0.0 (0.0, 1.5)	0.5 (0.0, 2.5)	0.6 (0.0, 2.6)	1.3 (0.0, 2.4)	0.328
DGLOV	0.0 (0.0, 0.0)	0.0 (0.0, 0.0)	0.0 (0.0, 0.0)	0.0 (0.0, 0.0)	0.402
Total rice & alternatives	10.0 (7.7, 10.0) ^b^	10.0 (8.3, 10.0) ^b^	10.0 (8.0, 10.0) ^b^	8.5 (7.2, 10.0) ^a^	0.007
Whole grains	0.0 (0.0, 0.0)	0.0 (0.0, 0.0)	0.0 (0.0, 0.0)	0.0 (0.0, 0.0)	0.581
Dairy	2.6 (0.0, 5.4) ^b^	2.1 (0.0, 7.0) ^b^	2.9 (0.0, 9.4) ^b^	0.0 (0.0, 2.9) ^a^	0.008
Total protein foods	10.0 (7.8, 10.0)	10.0 (6.9, 10.0)	10.0 (5.8, 10.0)	10.0 (6.2, 10.0)	0.766
Total Fat	10.0 (8.6, 10.0)	10.0 (7.2, 10.0)	10.0 (9.8, 10.0)	10.0 (8.4, 10.0)	0.372
Saturated fat	9.7 (7.3, 10.0)	9.7 (7.0, 10.0)	10.0 (7.7, 10.0)	10.0 (7.0, 10.0)	0.476
Sodium	0.0 (0.0, 0.0)	0.0 (0.0, 0.0)	0.0 (0.0, 0.0)	0.0 (0.0, 0.0)	0.462
Total HEI-SGA score	47.5 (41.7, 55.2)	48.8 (40.1, 56.1)	49.1 (41.9, 59.7)	47.0 (40.1, 52.6)	0.470

* Based on independent samples, Kruskal-Wallis. Groups that do not share a superscript are significantly different from each other by post-hoc Bonferroni tests. DGLOV—dark green leafy and orange vegetables. BMI categories used in Singapore for this age group: “At risk of nutrient deficiency” < 18.5; “Heathy range” 18.5–22.9; “Moderate risk (of developing cardiovascular diseases) 23.0–27.0; “High risk” > 27.0 [17].

**Table 4:** Median component and total HEI-SGA values across all participants by ethnicity.

	Median (IQR) Component and Total Scores			
	Chinese (*n* = 257)	Indian (*n* = 134)	Malay (*n* = 145)	*p*-Value *
Total fruit	0.0 (0.0, 5.0) ^a^	0.0 (0.0, 3.5) ^b^	0.0 (0.0, 1.0) ^b^	<0.001
Whole fruit	0.0 (0.0, 5.0) ^a^	0.0 (0.0, 3.2) ^b^	0.0 (0.0, 0.0) ^b^	<0.001
Total vegetables	1.4 (0.0, 3.6) ^a^	0.3 (0.0, 1.5) ^b^	0.0 (0.0, 0.9) ^b^	<0.001
DGLOV	0.0 (0.0, 3.7) ^a^	0.0 (0.0, 0.0) ^b^	0.0 (0.0, 0.0) ^b^	<0.001
Total rice & alternatives	10.0 (8.0, 10.0)	10.0 (7.9, 10.0)	10.0 (7.4, 10.0)	0.458
Whole grains	0.0 (0.0, 0.0) ^a^	0.0 (0.0, 0.0) ^a,b^	0.0 (0.0, 0.0) ^b^	0.001
Dairy	2.2 (0.0, 7.0) ^a,b^	2.8 (0.0, 7.8) ^a^	0.0 (0.0, 5.3) ^b^	0.013
Total protein foods	10.0 (6.9, 10.0)	10.0 (6.6, 10.0)	10.0 (7.1, 10.0)	0.705
Total Fat	10.0 (9.7, 10.0) ^a^	10.0 (6.7, 10.0) ^b^	10.0 (6.0, 10.0) ^b^	0.001
Saturated fat	10.0 (8.1, 10.0) ^a^	9.5 (5.8, 10.0) ^b^	9.1 (6.4, 10.0) ^b^	<0.001
Sodium	0.0 (0.0, 0.0) ^b^	0.0 (0.0, 0.0) ^a^	0.0 (0.0, 0.0) ^a^	0.009
Total HEI-SGA score	52.4 (44.4, 60.4) ^a^	47.6 (38.5, 54.9) ^b^	44.4 (37.2, 50.2) ^c^	<0.001

* Based on independent samples, Kruskal-Wallis. Groups that do not share a superscript are significantly different from each other by post-hoc Bonferroni tests. DGLOV—dark green leafy and orange vegetables.

**Table 5:** Median component and total HEI-SGA values in Singaporean students attending different educational institutions.

	Median (IQR) Component and Total Scores			
Components	ITE (*n* = 100)	JC (*n* = 100)	POLY (*n* = 334)	*p*-Values *
Total fruit	0.0 (0.0, 3.2) ^b^	4.6 (0.0, 5.0) ^a^	0.0 (0.0, 3.2) ^b^	<0.001
Whole fruit	0.0 (0.0, 0.4) ^b^	4.2 (0.0, 5.0) ^a^	0.0 (0.0, 4.0) ^b^	<0.001
Total vegetables	0.7 (0.0, 2.6) ^b^	2.7 (0.0, 5.0) ^a^	0.0 (0.0, 1.5) ^b^	<0.001
DGLOV	0.0 (0.0, 0.0) ^b^	0.0 (0.0, 3.7) ^a^	0.0 (0.0, 0.0) ^b^	<0.001
Total rice & alternatives	10.0 (6.3, 10.0) ^b^	10.0 (8.8, 10.0) ^a^	10.0 (7.9, 10.0) ^a,b^	0.015
Whole grains	0.0 (0.0, 0.0) ^a^	0.0 (0.0, 0.0) ^b^	0.0 (0.0, 0.0) ^b^	0.004
Dairy	0.0 (0.0, 4.0) ^b^	3.4 (0.0, 8.4) ^a^	2.1 (0.0, 7.3) ^a,b^	0.002
Total protein foods	10.0 (6.7, 10.0)	10.0 (7.6, 10.0)	10.0 (6.4, 10.0)	0.433
Total Fat	10.0 (9.8, 10.0)	10.0 (9.4, 10.0)	10.0 (7.0, 10.0)	0.034
Saturated fat	10.0 (8.6, 10.0) ^b^	10.0 (7.7, 10.0) ^a,b^	9.7 (6.5, 10.0) ^a^	0.008
Sodium	0.0 (0.0, 0.0)	0.0 (0.0, 0.0)	0.0 (0.0, 0.0)	0.788
Total HEI-SGA score	47.4 (40.2, 52.6) ^b^	56.6 (48.1, 64.4) ^a^	47.4 (38.2, 54.7) ^b^	<0.001

* Based on independent samples, Kruskal-Wallis. Groups that do not share a superscript are significantly different from each other by post-hoc Bonferroni-corrected tests. ITE—Institute of Technical Education, JC—Junior College, POLY—polytechnic, DGLOV–dark green leafy and orange vegetable.

## Appendix A

**Table A1:** Strengthening the reporting of observation studies in epidemiology (STROBE) checklist of recommended items that should be included in reports of cross-sectional studies.

Section	Item	Recommendation	Reported in
Title and abstract	1	(a) Indicate the study’s design with a commonly used term in the title or the abstract	Title and Abstract (p. 1 lines 19–20)
		(b) Provide in the abstract an informative and balanced summary of what was done and what was found	Abstract (p. 1)
Introduction			
Background/rationale	2	Explain the scientific background and rationale for the investigation being reported	Introduction (p. 1–2)
Objectives	3	State specific objectives, including any prespecified hypotheses	Objectives p. 1, lines 71–73. No prespecified hypotheses included.
Methods			
Study design	4	Present key elements of the study design early in the paper	Abstract, Introduction, and Methods
Setting	5	Describe the setting, locations, and relevant dates, including periods of recruitment, exposure, follow-up, and data collection	Exposure and follow-up not relevant to study design. Recruitment data collection dates included (Methods, p. 3, line 97).
Participants	6	(a) Give the eligibility criteria and the sources and methods of selection of participants	Methods, p. 3, lines 81–83)
Variables	7	Clearly define all outcomes, exposures, predictors, potential confounders, and effect modifiers. Give diagnostic criteria, if applicable	Outcomes defined throughout Methods section. Unmeasured potential confounders discussed (Discussion, line 256–262 and lines 269–272). Exposures and diagnostic criteria not applicable to current study.
Data sources/measurement	8	For each variable of interest, give sources of data and details of methods of assessment (measurement). Describe comparability of assessment methods if there is more than one group	Presented throughout the Methods section
Bias	9	Describe any efforts to address potential sources of bias	Methods, particularly lines 85–96
Study size	10	Explain how the study size was arrived at	Only an estimate of the adequacy of total population was considered, with broader convenience sampling based on available study timeline (Methods, lines 97–101)
Quantitative variables	11	Explain how quantitative variables were handled in the analyses. If applicable, describe which groupings were chosen and why	Data handling described in Methods (lines 86–128)
Statistical methods	12	(a) Describe all statistical methods, including those used to control for confounding variables	Methods (lines 129–134)
		(b) Describe any methods used to examine subgroups and interactions	Methods and Results
		(c) Explain how missing data were addressed	Not applicable (see Results line 136)
		(d) If applicable, describe analytical methods taking account of sampling strategy	Not carried out
		(e) Describe any sensitivity analyses	Not carried out. Potential unmeasured confounders discussed
Results			
Participants	13	(a) Report numbers of individuals at each stage of study—e.g., numbers potentially eligible, examined for eligibility, confirmed eligible, included in the study, completing follow-up, and analysed	No data collected on potential eligibility or number of individuals who declined to take part.
		(b) Give reasons for non-participation at each stage	Not applicable (see 13 (a)).
		(c) Consider use of a flow diagram	Not applicable (see 13 (a))
Descriptive data	14	(a) Give characteristics of the study participants (e.g., demographic, clinical, social) and information on exposures and potential confounders	Results Table 1, Table 2, Table 3, Table 4 and Table 5
		(b) Indicate the number of participants with missing data for each variable of interest	Not applicable (see 13 (a))
Outcome data	15	Report numbers of outcome events or summary measures	Results Table 1, Table 2, Table 3, Table 4 and Table 5
Main results	16	(a) Give unadjusted estimates and if applicable, confounder-adjusted estimates and their precision (e.g., 95% confidence interval). Make clear which confounders were adjusted for and why they were included	Confounder-adjusted estimates not applicable to the current study design
		(b) Report category boundaries when continuous variables were categorized	BMI categories used noted in Results (Table 3, lines 159–161).
		(c) If relevant, consider translating estimates of relative risk into absolute risk for a meaningful time period	Not applicable
Other analyses	17	Report other analyses done—e.g., analyses of subgroups and interactions and sensitivity analyses	Analyses of sub-groups described throughout Results. Interaction and sensitivity analyses not carried out.
Discussion			
Key results	18	Summarise key results with reference to study objectives	Discussion (lines 191–257)
Limitations	19	Discuss limitations of the study, taking into account sources of potential bias or imprecision. Discuss both the direction and magnitude of any potential bias	Discussion (lines 241–250, lines 258–264, lines 269–274, lines 275–301)
Interpretation	20	Give a cautious overall interpretation of results considering objectives, limitations, multiplicity of analyses, results from similar studies, and other relevant evidence	Major interpretations of results presented in lines 317–318 of Conclusions.
Generalisability	21	Discuss the generalisability (external validity) of the study results	Considered in relation to broader limitations of the study design (lines 275–301). Conclusions related to this are presented on lines 318–322
Other information			
Funding	22	Give the source of funding and the role of the funders for the present study and if applicable, for the original study on which the present article is based	Presented post-Conclusions (lines 329–330)
